# Gibberellins involved in fruit ripening and softening by mediating multiple hormonal signals in tomato

**DOI:** 10.1093/hr/uhad275

**Published:** 2023-12-18

**Authors:** Mengbo Wu, Kaidong Liu, Honghai Li, Ying Li, Yunqi Zhu, Dan Su, Yaoxin Zhang, Heng Deng, Yikui Wang, Mingchun Liu

**Affiliations:** Key Laboratory of Bio-Resource and Eco-Environment of Ministry of Education, College of Life Sciences, Sichuan University, Chengdu, 610065, Sichuan, China; Life Science and Technology School, Lingnan Normal University, Zhanjiang, 524048, China; Sichuan Academy of Forestry, Chengdu, 610081, Sichuan, China; Key Laboratory of Bio-Resource and Eco-Environment of Ministry of Education, College of Life Sciences, Sichuan University, Chengdu, 610065, Sichuan, China; Key Laboratory of Bio-Resource and Eco-Environment of Ministry of Education, College of Life Sciences, Sichuan University, Chengdu, 610065, Sichuan, China; Key Laboratory of Bio-Resource and Eco-Environment of Ministry of Education, College of Life Sciences, Sichuan University, Chengdu, 610065, Sichuan, China; Key Laboratory of Bio-Resource and Eco-Environment of Ministry of Education, College of Life Sciences, Sichuan University, Chengdu, 610065, Sichuan, China; Key Laboratory of Bio-Resource and Eco-Environment of Ministry of Education, College of Life Sciences, Sichuan University, Chengdu, 610065, Sichuan, China; Institute of Vegetable Research, Guangxi Academy of Agricultural Sciences, Nanning, 530007, Guangxi, China; Key Laboratory of Bio-Resource and Eco-Environment of Ministry of Education, College of Life Sciences, Sichuan University, Chengdu, 610065, Sichuan, China

## Abstract

The phytohormone ethylene is well known for its important role in the ripening of climacteric fruit, such as tomato (*Solanum lycopersicum*). However, the role and mode of action of other plant hormones in climacteric fruit ripening regulation are not fully understood. Here, we showed that exogenous GA treatment or increasing endogenous gibberellin content by overexpressing the gibberellin synthesis gene *SlGA3ox2* specifically in fruit tissues delayed tomato fruit ripening, whereas treatment with the GA biosynthesis inhibitor paclobutrazol (PAC) accelerated fruit ripening. Moreover, exogenous ethylene treatment cannot completely reverse the delayed fruit ripening phenotype. Furthermore, exogenous GA treatment of ethylene signalling mutant *Never ripe* (*Nr*) or *SlEBF3*-overexpressing lines still delayed fruit ripening, suggesting that GA involved in fruit ripening partially depends on ethylene. Transcriptome profiling showed that gibberellin affect the ripening of fruits by modulating the metabolism and signal transduction of multiple plant hormones, such as auxin and abscisic acid, in addition to ethylene. Overall, the results of this study provide new insight into the regulation of gibberellin in fruit ripening through mediating multiple hormone signals.

## Introduction

Tomato is a crucial agricultural commodity globally and plays an important role in the balanced human diet. It is abundant in a variety of nutrients, such as carotenoids, flavonoids, organic acids, and vitamins, which can help in the prevention of different types of cancers and cardiovascular ailments [1]. Tomato has been widely adopted as a model system for investigating the control of fruit development and ripening due to its relatively short life cycle, moderate genome size (approximately 900 Mb) and ease of genetic transformation.

The maturation of tomato fruit is an intricate, synchronized developmental procedure that encompasses a range of transformations, including changes in colour, flavour, nutrition, and firmness [2–4]. Among all the changes, colour turning caused by the breakdown of chlorophyll and the accumulation of carotenoids and softening resulting from cell wall degradation are the two most common signs of fruit ripening [2, 4]. For a long time, it has been recognized that ethylene plays a crucial role in starting and advancing the ripening process of tomatoes [5, 6]. Manipulation of exogenous or endogenous ethylene content alters the ripening process of fruits [1, 7, 8]. Moreover, a number of key transcription factors, such as RIPENING INHIBITOR (RIN), NON-RIPENING (NOR), FRUITFULL 1 (FUL1), TOMATO AGAMOUS-LIKE1 (TAGL1), AP2a, and SlERF.F12, have been reported as regulators of tomato fruit ripening. These factors control the expression of genes involved in ethylene production, carotenoid synthesis, and cell wall breakdown [4, 9–18]. Recent studies have shown that fruit ripening is also influenced by epigenetic changes such as DNA methylation, RNA methylation, and histone acetylation and methylation [1, 4, 19–21]. It is worth mentioning that all known regulators responsible for the ripening process in tomatoes thus far control the ripening of fruits in a manner that relies on ethylene, indicating the crucial involvement of ethylene in fruit ripening.

In addition to ethylene, increasing evidence has shown that other plant hormones, such as abscisic acid (ABA), auxin, and brassinosteroids (BRs), also play important roles in the process of fruit maturation [22–28]. The application of external ABA can induce the production of ethylene and accelerate the ripening process, including softening [22]. Inhibiting the transcription of genes that encode major cell wall catabolic enzymes delays the fruit ripening process by reducing the endogenous ABA content through downregulation of 9-*cis*-epoxycarotenoid dioxygenase (SlNCED), a crucial gene involved in ABA biosynthesis [29]. The silencing of *SlNCED1* by VIGS inhibited ABA accumulation and fruit ripening in tomato [30]. These results indicate that ABA coordinates with ethylene to promote fruit ripening. Auxin plays a role in the maturation of fruits in a manner that relies on ethylene [31–33]. It was shown that auxin inhibits the ripening process by maintaining system I ethylene production and inhibiting system II ethylene bursts [32]. The inhibition of fruit ripening and ethylene production, along with the decreased expression of ethylene biosynthesis genes, was observed when *SlARF2A* and *SlARF2B* were silenced using RNAi [34]. It has been reported that the *SlSAUR69* gene, which responds to auxin, influences the ripening of fruits by affecting ethylene sensitivity [33]. Moreover, recent studies have demonstrated that BRs play a role in the regulation of fruit ripening by interacting with ethylene [26, 27]. Furthermore, in addition to these plant hormones, gibberellins (GAs) have also been shown to be involved in the fruit ripening process [35–37]. Nevertheless, the complete understanding of the underlying process by which GAs affect the fruit ripening remains elusive.

GA is an important plant hormone involved in various stages of plant development, encompassing seed sprouting, root expansion, elongation of hypocotyls and stems, flowering, fruit setting, and the growth of fruits [38–40]. Because GA content mainly accumulates during early fruit development [41], previous research has primarily focused on the impact of GA on tomato fruit set and development. The application of PAC, a GA biosynthesis inhibitor, decreases the rate of tomato fruit set and hinders fruit growth. However, this effect can be reversed by applying endogenous GAs [42, 43]. Inhibition of GA4 synthesis, which is one of the bioactive forms of GAs, led to a notable decrease in fruit set observed in the tomato tap3 mutant [44]. While the role of GAs has mainly been studied in fruit set and development, research has demonstrated their crucial role as regulators in fruit ripening. It was shown that GA treatment retarded ripening in terms of the development of redness [35]. Moreover, a recent investigation indicated that the application of GA treatment can postpone the ripening of tomato fruits through repression of the expression of genes involved in ethylene synthesis and signal transduction, as well as the expression of ripening regulator genes such as RIN, NOR, and CNR [36]. More recently, it was reported that GA delayed fruit ripening by promoting auxin signalling [37]. These studies suggested that GA may affect both ethylene and auxin signalling; however, whether GA modulates other plant hormones, such as ABA and BRs, during fruit ripening remains unclear.

In the present study, we examined the role of GAs in the maturation of fruits and found that GAs can interact with ethylene and auxin to affect fruit ripening, which is consistent with previous studies [36, 37]. Our results suggest that gibberellins can also interact with multiple hormonal signals, such as abscisic acid and brassinosteroids, to coordinate the ripening process. Moreover, we showed that GAs suppress fruit ripening by affecting epigenetic modifications such as DNA methylation. Our results thus provide new perspectives on how GA influences the process of fruit ripening.

## Results

### GA content decreased during tomato fruit ripening

**Figure 1:**
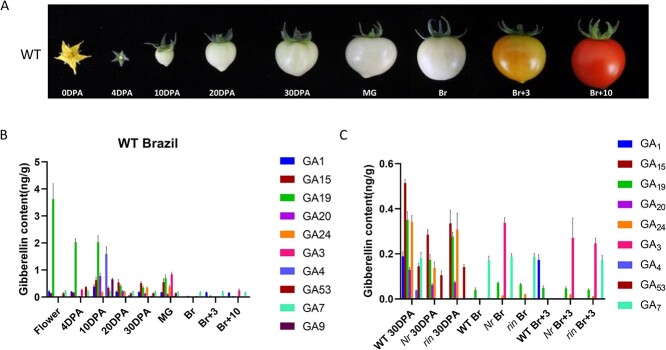
Gibberellin content decreased during fruit ripening. **A** The different stages of tomato fruit. **B** Concentrations of GA in wild type tomato fruit at different stages analysed by UPLC-ESI-MS/MS. GA content showed a high level in fruit development stage. Endogenous GA gradually decreased during fruit ripening. **C** The GA content of Nr and rin mutants at various stages. Br, breaker stage; DPA, days post anthesis; MG, mature green stage.

To investigate the potential function of GAs in the ripening of fruits, we quantified the endogenous GA content in both flowers and fruits at various stages of development and ripening in tomato (Fig. 1A and B). The total GA content in flowers and fruits was relatively high during developmental stages, but it significantly decreased when ripening began (from the MG to Br stage). It continued to remain at low levels throughout the ripening stages (Fig. 1B;Table S1, see online supplementary material). This result suggests a repression of GA during the process of fruit maturation. To further validate the function of GA in the ripening of fruits, we also monitored the GA levels in two tomato mutants associated with ripening, namely, *ripening inhibitor* (rin) and *Never ripe* (Nr). Interestingly, the results showed that at the Br and Br + 3 stages, the content of total GA, especially GA3, an active form of GA, was notably elevated in the two ripening mutants compared with WT, while the total content of GA was comparable at the unripe stage (30 DPA) (Fig. 1C; Table S2, see online supplementary material), indicating a negative role of GA in the process of fruit ripening. Taken together, these results indicate that the ripening process coincides with a gradual loss of GA, and it is reasonable to consider that GA inhibits fruit ripening.

### Exogenous application of GA delays fruit ripening and softening

**Figure 2:**
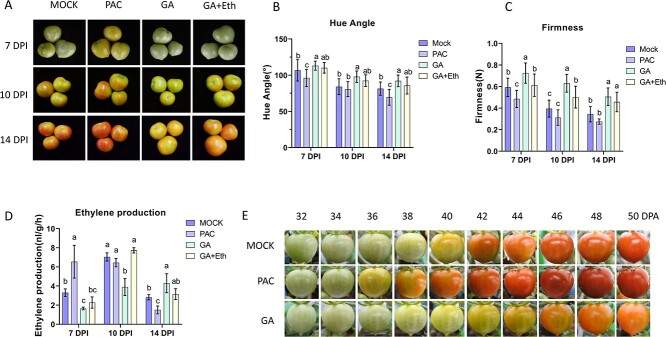
Gibberellins (GAs) suppress tomato fruit ripening. **A** The fruit ripening process after different treatments. Fruit collected at the immature green stage (32 DPA) were treated with GA or PAC, and stored at 22°C, and 3 days later, one of the GA-treated fruits was treated again with ethylene. **B–D** Hue angle **(B)**, fruit firmness **(C)**, and ethylene production **(D)** after different treatments. The data represent means ± SDs of ten biological replicates. Significant differences were determined using the ANOVA test, with different letters representing statistical significance (*P* < 0.05). **E** The process of fruit ripening after different treatments on the vine. GA, fruit treated with GA；GA + Eth, fruits treated with GA and ethylene; Mock, fruit treated with distilled water; PAC, fruit treated with PAC.

To assess the influence of GA on fruit ripening, we treated the fruits with GA and PAC, a GA synthesis inhibitor, at 32 DPA and then added ethylene to GA-treated fruits at 35 DPA. Compared with the control (mock), ripening of GA-treated fruits was significantly delayed but promoted in the PAC-treated fruit (Fig. 2A). As shown in Fig. 2A, when the mock fruits started to reach the Br stage (7 days post injection (DPI) of the respective treatments), the fruits treated with PAC were at the post Br stage, but the fruit samples treated with GA or GA + ethylene (Eth) were still at the MG stage. This suggests that GA has a detrimental effect on fruit ripening. At both 10 and 14 DPI, the ripening process was repressed in GA-treated fruits compared with the mock and PAC-treated fruits (Fig. 2A). Interestingly, ethylene treatment failed to fully reverse the ripening inhibition induced by GA (Fig. 2A), suggesting that ripening inhibition by GA might be partially dependent on ethylene.

Fruit colour, firmness, and ethylene production were also measured as indicators of the fruit ripening process (Fig. 2B–D). During storage after treatment, the hue angle value of each treatment group decreased gradually, indicating that the tomato fruit gradually turned red (Fig. 2B). The hue angle value of the fruit treated with GA was consistently higher than that of the mock control, whereas the PAC-treated fruit generally exhibited lower values, particularly at 14 DPI (Fig. 2B). The colour change of the GA-treated fruit was the slowest, as was the decrease in the hue angle value (Fig. 2B). At all three time points, there was no statistical distinction in the hue angle value between the GA + Eth fruit and the mock fruit. The difference in firmness due to different treatments was highly significant. Fruit treated with PAC softened earlier than fruit treated with GA with or without ethylene (Fig. 2C). At 10 DPI, GA + Eth-treated tomatoes were significantly firmer than those of the mock group (Fig. 2C), which indicated that ethylene treatment could not fully compensate for the inhibitory effect of GA on fruit softening. Consistent with the fruit colour change and softening, at 7 and 10 DPI, the GA-treated fruits produced less ethylene than the other fruits (Fig. 2D). At 14 DPI, when the ethylene production of the mock group decreased, the ethylene production of GA-treated fruits increased (Fig. 2D). In comparison with the mock group, ethylene did not completely reverse the effect of GA on fruit ripening in the GA + Eth-treated fruit (Fig. 2D). The above results showed that gibberellin inhibits fruit ripening, while exogenous ethylene only partially compensates for the effect of inhibition of gibberellin on fruit ripening. The potential impact of gibberellin on the maturation of fruit might therefore not be completely dependent on ethylene action.

To further validate the impact of GA on fruit ripening, we also treated fruit on the vine with injected GA and PAC. The results showed that GA treatment still delayed fruit ripening, while PAC treatment made the fruit ripen earlier (Fig. 2E), which is consistent with the off-vine treatment results.

### Exogenous GA treatment extends the shelf life of tomato fruit

**Figure 3:**
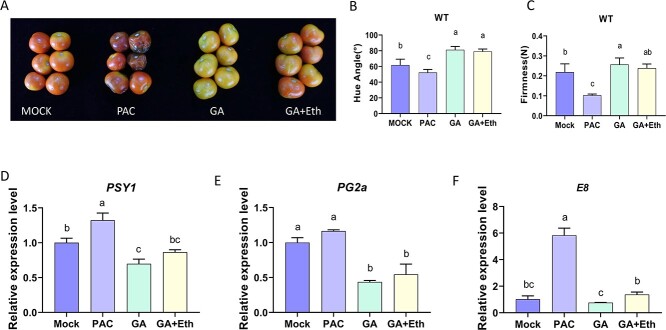
Gibberellin treatment affects the shelf life of tomato fruit. **A** Fruit phenotype after 30 DPI with different treatments. The photo was taken after 30 DPI. GA + Eth, fruits treated with GA and ethylene. **B–C** Hue angle **(B)** and firmness **(C)** of fruit after the treatment. Ten biological replicates were analysed. **(D)** RT–qPCR analysis of *PSY1* transcript levels after different treatments. **E** RT–qPCR analysis of *PG2*a transcript levels. **F** RT–qPCR analysis of *E8* transcript levels. The mRNA levels of each gene in the mock group were standardized to 1 and normalized with actin genes. The data represent means ± SDs of three biological replicates. Statistical significance was determined using ANOVA test (*P* < 0.05).

To further examine the impact of GA on the softening of fruits and their shelf life, we stored the fruits under different treatments at room temperature until 30 DPI. The degree of fruit softening between different treatments was visibly different (Fig. 3A). The GA-treated tomatoes were the least ripe and remained firm with peels that were still mostly yellow. The pericarp of the fruits treated with GA and ethylene was orange–red even after 30 DPI. In marked contrast, the tomato fruit treated with PAC was almost decayed. These results show that the amount of gibberellin, whether endogenous or exogenous, strongly influences the shelf life of tomatoes. Other quantifiable indicators of fruit ripening confirmed this observation. In Fig. 3B, the hue angle of PAC-treated fruit, which had inhibited GA biosynthesis, was notably lower than the hue angles of the other three groups at 30 DPI. However, the hue angles of fruit treated with GA or GA + Eth were considerably higher than the hue angles of the mock control fruit (Fig. 3B). The fruit that received GA treatment were firmer than mock fruit, while fruit treated with PAC were significantly softer than the other three groups (Fig. 3C). RT–qPCR was performed to detect the expression changes of maturation-associated genes. The RT–qPCR results showed that the transcript levels of the crucial gene PSY1 related to carotenoid production, the gene PG2a associated with softening, and the gene E8 responsive to ethylene were notably reduced in GA-treated fruit compared to the mock and PAC-treated fruits, which is consistent with the fruit colour and softening phenotypes (Fig. 3D–F).

### Increasing endogenous GA content by overexpression of *SlGA3ox2* represses fruit ripening in tomato

**Figure 4:**
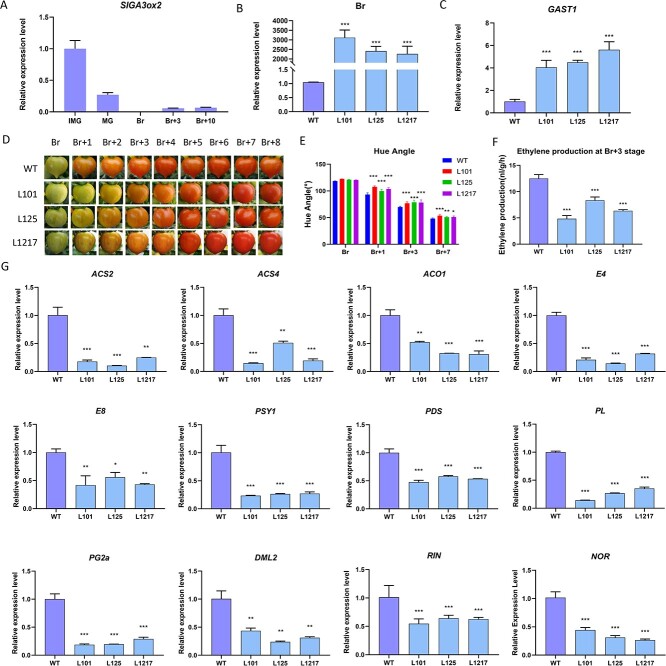
The fruit ripening process of GA3ox2 overexpression plants is affected. **A** The expression pattern of GA3ox2 during tomato fruit ripening process. **B** RT–qPCR analysis of SlGA3ox2 expression level in fruits in WT and GA3ox2-OE lines at the Br stage. **C** SlGAST1 relative expression levels in GA3ox2-OE breaker stage fruits. **D** Photograph of WT and GA3ox2 overexpression plants (L101, L125, L1217) at different stages. **E** Hue angle value of WT and GA3ox2-OE fruits during fruit ripening. **F** Ethylene production in WT and GA3ox2-OE fruits during fruit ripening stages. **G** Expression of the genes related to maturation at Br stage. The data are shown as means ± SD of at least three biological replicates. (Student’s *t* test, ^*^P < 0.05 and ^**^P < 0.01.)

To explore the impact of endogenous gibberellin on the maturation of fruits, we focused on *SlGA3ox2*, a gibberellin synthesis gene whose product is a hydroxylase that catalyses the production of active GA from inactive precursors. Because *SlGA3ox2* exhibits low expression levels during fruit ripening (Fig. 4A), we used the promoter of *E8*, which is specific for fruit ripening, to overexpress *SlGA3ox2* in tomato. We first confirmed that *SlGA3ox2* was overexpressed in transgenic fruit at the break (Br) stage (Fig. 4B) and that the expression level of SlGAST1, a GA-responsive gene, was increased (Fig. 4C). Moreover, the expression level of *SlGA3ox2* in leaves was not different between WT and *SlGA3ox2*-OE lines (Fig S1, see online supplementary material). The colour change of the transgenic fruit after the Br stage was slower than that of the wild-type fruit (Fig. 4D and E). Monitoring ethylene production showed that *SlGA3ox2*-OE fruits produced less ethylene than WT fruits at the Br + 3 stage (Fig. 4F). To study how increased *SlGA3ox2* expression affects fruit maturation, we analysed the transcript levels of the genes related to maturation at the Br stage. In *SlGA3ox2*-OE fruits, there was a notable reduction in the levels of *ACS2, ACS4, ACO1, E4,* and *E8*, which are crucial genes responsible for ethylene production or the ethylene response, as observed in the comparison with WT (Fig. 4G). The relative expression of *PSY1* and *PDS*, two key genes associated with carotenoid synthesis, was also decreased in *SlGA3ox2*-OE fruits at the Br stage (Fig. 4G). In the fruits of the SlGA3ox2-OE lines, the transcription of genes associated with fruit softening, such as PL and PG2a, showed a significant decrease (Fig. 4G). In addition, *SlDML2*, a key gene controlling fruit ripening by affecting DNA methylation, displayed decreased expression levels in *SlGA3ox2*-OE lines (Fig. 4G). Moreover, the expression levels of *RIN* and *NOR*, two key ripening regulators, were decreased in *SlGA3ox2*-OE fruits at the Br stage (Fig. 4G). These results support the assumption that an increase in endogenous gibberellin content in fruits through overexpression of *SlGA3ox2* inhibited the ripening process.

### GA inhibits the maturation procedure in ethylene signal-impaired tomato mutants

**Figure 5:**
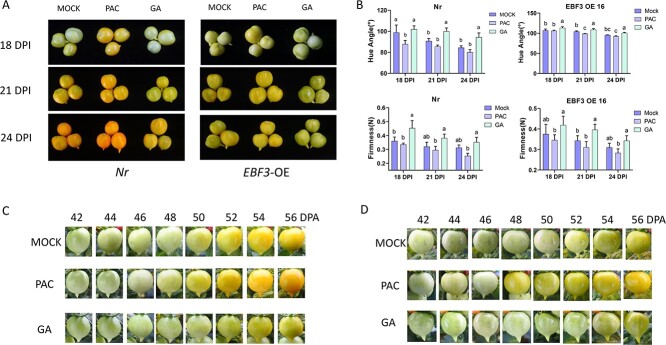
Exogenous GA-treatment inhibits fruit ripening process in ethylene signal impaired mutants**. A** Fruits treated with GA exhibited a postponed ripening phenotype in Nr (left) and EBF3-DE (right). DPI, days post injection. **B** GA-treatment affected the fruit hue angle and firmness compared to control fruits in Nr (left) and EBF3-OE (right) plants. The data are shown as means ± SD of at least ten biological replicates. Univariate ANOVA tests were used to analyse the significance of differences among treatment groups. **C–D** GA inhibited the fruit ripening process on the vine in Nr (**C**) and EBF3-OE (**D**) plants.

As ethylene treatment did not completely reverse the effects of GA on fruit ripening, we speculated that GA affects some aspects of fruit ripening by acting through an ethylene-independent pathway. To confirm whether the suppressive impact of GA on fruit maturation relies partly on ethylene, we subjected fruit maturation-deficient mutant Nr (Never ripe) and EBF3-OE transgenic lines, both exhibiting impaired ethylene signalling but via distinct mechanisms [45, 46], to treatment with PAC or GA with or without ethylene at 32 DPA. After exogenous GA treatment, compared with the mock, both *Nr* and *EBF3-OE* fruits exhibited a postponement in fruit ripening, while PAC treatment advanced the ripening process (Fig. 5A). The fruit treated with GA had higher firmness and hue angle values than the mock and PAC-treated fruits (Fig. 5B). The inhibitory effect of GA on ripening in the ethylene signalling-impaired ripening mutants *Nr* and *EBF3-OE* suggested that GA represses fruit ripening at least partially independent of ethylene. Moreover, the impact of GA and PAC on fruit ripening was tested on the vine, and the result was consistent with the off-vine treatment (Fig. 5C and D). These results indicated that GA inhibited the fruit ripening process in ethylene signal-impaired mutants.

### Gibberellin treatment alters multiple pathways related to ripening traits and phytohormones

**Figure 6:**
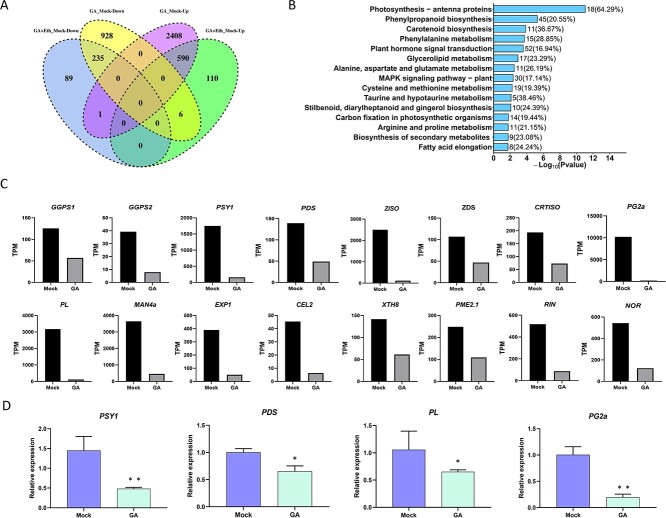
RNA-seq profiling of tomato fruits with different treatments. **A** Venn diagram of differentially expressed genes (DEGs). The intersection of downregulated and upregulated genes that exhibited differential expression under GA and GA + Eth treatment compared to mock treatment. **B** KEGG analyses of DEGs between mock and GA treatment. **C** Expression levels of carotenoids and softening-related genes in transcripts per million. **D** RT–qPCR analysis of the expression level of genes related to ripening. The mRNA levels of each gene in the mock sample were standardized to 1. The error bars represent the standard errors.

To obtain a deeper understanding of the impact of GA on fruit ripening at the molecular level, we conducted transcriptome analysis using RNA sequencing (RNA-seq) on fruits treated with GA and GA + Eth at 10 DPI. The high-quality reads obtained from each sample were counted, and DESeq2 was used to identify the upregulated or downregulated differentially expressed genes in the GA and GA + Eth samples compared to the mock samples (Tables S3 and S4, see online supplementary material). The results are presented in the form of a Venn diagram (Fig. 6A). Compared with mock-treated fruit samples, 1169 genes were downregulated under the influence of GA alone, while 325 genes were downregulated under GA plus ethylene treatment (Fig. 6A). In addition, 2999 genes were upregulated under GA alone compared with mock-treated fruit samples, while only 706 genes were upregulated under GA plus ethylene (Fig. 6A). These results suggested that a high ratio of GA repressed or activated genes can be recovered by ethylene. Interestingly, as shown in Fig. 6A, 825 genes, including 235 downregulated genes (intersection of GA_Mock_Down and GA + Eth_Mock_Down) and 590 upregulated genes (intersection of GA_Mock_Up and GA + Eth_Mock_Up), were affected by GA at least partially in an ethylene-independent manner.

**Figure 7:**
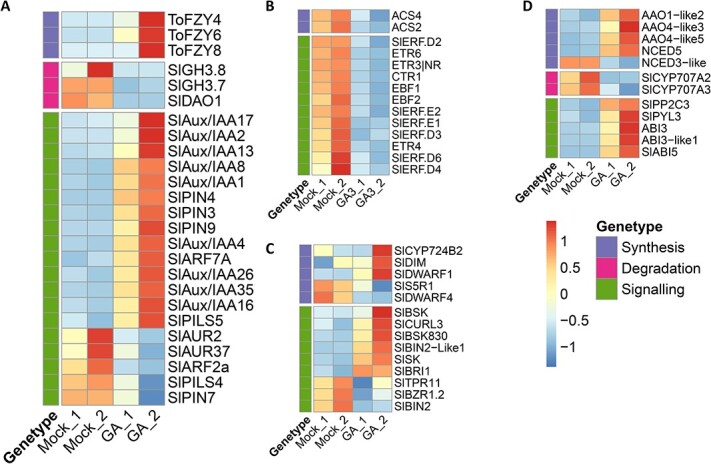
Heatmap showing the impact of GA treatment on the expression of hormone-related genes. The overview of main hormone DEGs related to synthesis, degradation, and signalling for auxin (**A**), ethylene (**B**), brassinosteroid (**C**), abscisic acid (**D**).

According to the KEGG pathway enrichment analysis, the pathways associated with photosynthesis, phenylpropane biosynthesis, carotenoid biosynthesis, plant hormone signal transduction, and phenylalanine metabolism were found to be the most significantly enriched in response to GA (Fig. 6B), suggesting that ripening-related processes were affected by GA treatment. Furthermore, the downregulation of genes involved in carotenoid production (*GGPS1, GGPS2, PSY1, PDS, ZISO, ZDS*, and *CRTISO*) and genes associated with fruit softening (*PG2a, PL, MAN4a, EXP1, CEL2, XTH8,* and *PME2.1*) together with the RT–qPCR results (Fig. 6C and D) further supported the repressive function of GA in the regulation of fruit ripening. Interestingly, the expression levels of a number of genes related to the production or signal transduction of plant hormones, including auxin, ethylene, ABA, and BRs, were found to be altered in GA-treated fruits (Fig. 7A–D). In particular, genes related to auxin, which is known to act as a repressor in ripening, were mostly upregulated. Conversely, a majority of genes linked to ethylene were downregulated in GA-treated fruits compared to the control. These results suggested that GA repressed fruit ripening by affecting multiple plant hormones in addition to ethylene.

### GA inhibits fruit ripening partially independent of ethylene

**Figure 8:**
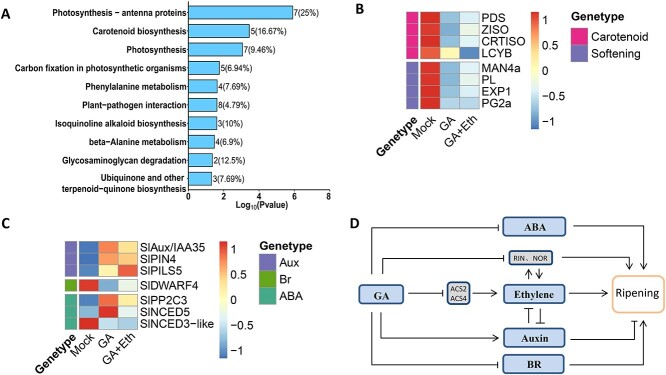
GA affect ripening process partially independent on ethylene. **A** KEGG analysis of DEGs affected by GA and GA + Eth (including 235 downregulated genes and 590 upregulated genes). **B** The expression of carotenoid and softening-related genes after different treatment. **C** The alteration of genes associated with hormones following various treatments. GA, fruit treated with GA；GA + Eth, fruits treated with GA and ethylene; Mock, fruit treated with distilled water; PAC, fruit treated with PAC. **D** The working model of GA in modulation of fruit ripening.

To further examine the control of GA on fruit maturation, we performed KEGG pathway enrichment analysis on the 825 genes (235 downregulated genes and 590 upregulated genes) that were regulated by GA at least partially in an ethylene-independent manner and found that pathways including carotenoid biosynthesis and photosynthesis were among the pathways that were enriched (Fig. 8A). Moreover, the transcript levels of several crucial genes related to carotenoid biosynthesis (*PDS, CRTISO, ZISO*, and *b-LCY*) and cell softening-related genes (*PL, PG2a, EXP1*, and *MAN4a*) could not be fully recovered by GA + Eth treatment compared with GA treatment (Fig. 8B). In addition, the transcript levels of certain genes related to auxin, Br, or ABA also fail to be recovered by ethylene treatment (Fig. 8C), supporting the notion that the suppressive impact of GA on the maturation of fruits is partially independent of ethylene.

## Discussion

Without minimizing the essential role of ethylene, an increasing amount of evidence indicates that other plant hormones also have a major impact on the ripening of climacteric fruits. Among these plant hormones, GA is also believed to play an important role in modulating the process of fruit ripening [35, 36]. However, the precise regulatory mechanism of how GA represses fruit ripening is not well understood. In particular, whether the regulation is fully dependent on ethylene remains elusive. In this study, we validated the repressive effect of GA on fruit ripening through exogenous GA treatment and increased the endogenous GA content via overexpression of a GA biosynthesis gene (*SlGA3ox2*) and revealed that GA represses ripening traits partially independent of ethylene. The results of our study not only provide new insight into the regulation of plant hormones in fruit ripening but also shed light on the regulatory network of climacteric fruit ripening.

Studies have demonstrated that GA represses fruit ripening by affecting the production of ethylene and/or the transmission of signals in tomato [36]. During our investigation, we found that the addition of ethylene to GA-treated wild-type fruits failed to fully restore the ripening inhibition phenotype, including fruit colour, firmness and the expression levels of some key ripening genes. This suggested that GA represses fruit ripening and may not be fully dependent on ethylene. Moreover, the inhibition of the ripening process by GA treatment and the promotion of ripening with PAC application in two ethylene signalling-impaired mutants supported the notion that GA regulation of fruit ripening might be partially independent of ethylene.

A number of studies have indicated that plant hormones affect plant growth and development in a crosstalk manner [29, 32, 47]. Our RNA-seq analysis data indicated that GA treatment not only affected the ethylene-associated pathways but also changed the pathways related to auxin, ABA, BR, and other plant hormones. Among these plant hormones, auxin has long been thought to play a role in the process of fruit ripening, and it was shown that auxin can repress the ripening of tomato fruit by influencing the levels of ethylene and ABA as well as ripening-related genes involved in lycopene biosynthesis and chlorophyll degradation [31]. Our study showed that the involvement of GA in the repression of fruit ripening may occur through the modulation of auxin metabolism and/or signal transduction since many genes related to auxin biosynthesis or signalling pathways were altered in GA-treated fruits, which is consistent with a recent report that GA delays metabolic shifts during fruit ripening by inducing auxin signalling in tomato [37]. In addition to auxin, the biosynthesis and/or signalling pathways of other plant hormones, such as ABA and BR, were also affected by GA treatment. Because both ABA and BR have been reported to play an important role in climacteric fruit ripening [26, 48–50], the alteration of these plant hormones may contribute to GA-mediated ripening inhibition. Moreover, because fruit ripening is a process mediated by the interaction of multiple plant hormones [51], our study supported that the repressive role of GA in controlling fruit ripening may be attributed to its impact on the production and/or signal transduction of other phytohormones and thus disrupting their interaction in the modulation of the ripening process.

Overall, we established a model to show the impact of GA on the modulation of fruit ripening (Fig. 8D). On the one hand, GA repressed the biosynthesis and signal transduction of ethylene by inhibiting the expression of genes related to ethylene synthesis and signalling. On the other hand, GA represses the ripening process of fruit by affect the metabolism and signal transduction of other plant hormones, such as auxin, ABA, and BRs. However, it is still unclear exactly how GA affect other plant hormones during the fruit ripening process, and further work will focus on illustrating the regulatory mechanism of GA in repressing fruit ripening.

## Materials and methods

### Plant materials and growth conditions

The Micro-Tom tomato cultivar (*Solanum lycopersicum*) was utilized for all experiments in this study. After being sterilized, the seeds were washed with sterile water. The seeds were germinated in 1/2 × Murashige and Skoog (MS) medium and grown on soil in a climate-controlled greenhouse as described by Deng *et al.* [45].

### Measurement of gibberellin content

WT tomato fruit tissues were collected at different stages. Nr and rin mutant fruit tissues were collected at 30 DPA, Br, and Br + 3. After removing any placenta or seed tissue from the harvested samples, they were promptly frozen in liquid nitrogen and stored at a temperature of −80°C. Tissue samples were ground to a dry powder. MetWare (http://www.metware.cn/) was used to measure the content of gibberellin in the samples. Each assay was performed in three replicates.

### Hormone and inhibitor treatments

The flowers were labelled on the day of pollination. Tomatoes at 32 days postanthesis (DPA) were selected for different treatments. The selected tomatoes were subjected to injection using a microsyringe with 100 ppm GA3 (Sigma, G7645, St Louis, MO, USA) and 100 ppm PAC (Sigma, P0115, St Louis, MO, USA). The injection was precisely targeted at the pericarp area in proximity to the sepal, with an approximate volume of 200 μL per fruit. After 3 days, some GA_3_-treated fruits were kept for 24 h in 150 mL sealed jars treated with 1000 ppm ethylene. Twelve fruits were used for each treatment. Treated fruits were stored in an artificial climate box. The fruit tissues were gathered and quickly frozen using liquid nitrogen, with the exclusion of the placenta and seeds.

### Fruit colour and firmness measurement

The colour and firmness of transgenic and wild-type (WT) tomato fruits were assessed at different stages. WT tomato fruits treated with GA, GA + Eth, or distilled water were analysed at 7, 10, and 14 DPI. Nr mutant and EBF3-OE tomato fruits treated with GA, GA + Eth, or distilled water were analysed at 18, 21, and 24 DPI. Fruit colour and firmness assessment was conducted following the methodology outlined by Deng *et al.* [45]. Sixteen fruits were measured for each genotype-treatment-stage category. Fruit firmness was evaluated by measuring the maximum force at two opposing points along the equator of the fruit. This value was then recorded as the representative measurement of fruit firmness. For each genotype-treatment-stage combination, five fruits were assessed.

### Ethylene measurement

Tomato fruits from a variety of stages and hormone treatments were collected and placed in sealed jars of 150 mL capacity for two hours. Subsequently, a syringe was used to extract 1 mL of headspace gas from each jar, which was then subjected to gas chromatography analysis [52]. Through comigration with an ethylene standard, ethylene was identified and quantified using a standard curve for ethylene concentration.

### RNA sequencing (RNA-seq) analysis

WT tomato fruits treated with GA, GA + Eth, or ethanol at 10 DPI were collected and promptly frozen using liquid nitrogen, excluding the placenta and seeds. Subsequently, these samples were stored at −80°C. Liquid nitrogen was used to grind the fruit pericarp into a fine powder. Illumina HiSeq 2000 was used to prepare and sequence the libraries. A data analysis was performed in accordance with Deng *et al.* [45]

### RNA extraction and qPCR

The fruit pericarp of different stages or different treatments was collected. Total RNA was isolated from the tissues using a plant RNA Extraction Kit (TaKaRa, Shiga, Japan, Code No. 9767). The Primerscript RT Reagent Kit (TaKaRa Code No. RR047A) with gDNA Eraser was used to reverse transcribe total RNA into cDNA. qPCR was carried out as described by Deng *et al.* [45]. Actin gene expression was used as an internal control. The primers for qPCR are listed in Table S5 (see online supplementary material).

### Plant transformation

The plasmid was transformed into Micro-Tom plants using *Agrobacterium tumefaciens* strain GV3101 by an *A. tumefaciens*-mediated procedure as described [53]. A medium containing kanamycin was used to select the transformed lines. All experiments were conducted using either the F2 or F3 generations.

## Supplementary Material

Web_Material_uhad275

## Data Availability

All relevant data and figures in this study can be found within the article and its supporting materials.
