# Supplementary figures and images for: Gibberellins involved in fruit ripening and softening by mediating multiple hormonal signals in tomato
Source: Hortic Res. 2023 Dec 18;11(2):uhad275. doi: 10.1093/hr/uhad275 (PMC10857933; doi:10.1093/hr/uhad275)

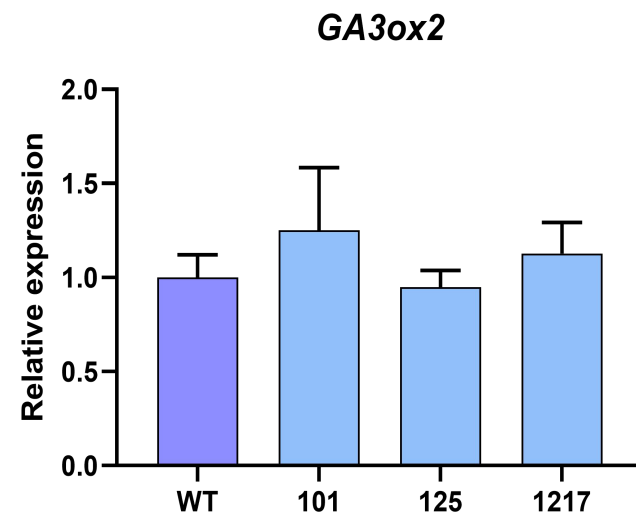

Supplement: Web_Material_uhad275 [file web_material_uhad275.zip › Supplement Fig S1.pdf]
